# Pathobiology of Type 2 Inflammation in Asthma and Nasal Polyposis

**DOI:** 10.3390/jcm12103371

**Published:** 2023-05-09

**Authors:** Corrado Pelaia, Giulia Pelaia, Angelantonio Maglio, Caterina Tinello, Luca Gallelli, Nicola Lombardo, Rosa Terracciano, Alessandro Vatrella

**Affiliations:** 1 Department of Health Sciences, University “Magna Græcia” of Catanzaro, 88100 Catanzaro, Italy; giulia.pelaia@gmail.com (G.P.); gallelli@unicz.it (L.G.); 2 Department of Medicine, Surgery and Dentistry, University of Salerno, 84084 Salerno, Italy; amaglio@unisa.it (A.M.); avatrella@unisa.it (A.V.); 3 Pediatrics Unit, Provincial Outpatient Center of Catanzaro, 88100 Catanzaro, Italy; tinello.caterina@gmail.com; 4 Department of Medical and Surgical Sciences, University “Magna Græcia” of Catanzaro, 88100 Catanzaro, Italy; nlombardo@unicz.it; 5 Department of Experimental and Clinical Medicine, University “Magna Græcia” of Catanzaro, 88100 Catanzaro, Italy; terracciano@unicz.it

**Keywords:** severe asthma, nasal polyposis, united airway diseases

## Abstract

Asthma and nasal polyposis often coexist and are frequently intertwined by tight pathogenic links, mainly consisting of the cellular and molecular pathways underpinning type 2 airway inflammation. The latter is characterized by a structural and functional impairment of the epithelial barrier, associated with the eosinophilic infiltration of both the lower and upper airways, which can be driven by either allergic or non-allergic mechanisms. Type 2 inflammatory changes are predominantly due to the biological actions exerted by interleukins 4 (IL-4), 13 (IL-13), and 5 (IL-5), produced by T helper 2 (Th2) lymphocytes and group 2 innate lymphoid cells (ILC2). In addition to the above cytokines, other proinflammatory mediators involved in the pathobiology of asthma and nasal polyposis include prostaglandin D_2_ and cysteinyl leukotrienes. Within this context of ‘united airway diseases’, nasal polyposis encompasses several nosological entities such as chronic rhinosinusitis with nasal polyps (CRSwNP) and aspirin-exacerbated respiratory disease (AERD). Because of the common pathogenic origins of asthma and nasal polyposis, it is not surprising that the more severe forms of both these disorders can be successfully treated by the same biologic drugs, targeting many molecular components (IgE, IL-5 and its receptor, IL-4/IL-13 receptors) of the type 2 inflammatory trait.

## 1. Introduction

Asthma and chronic rhinosinusitis with nasal polyps (CRSwNP) are widespread, often coexisting as inflammatory disorders affecting the lower and upper airways, respectively [1,2]. Asthma is usually characterized by shortness of breath, reversible airflow limitation, and bronchial hyperresponsiveness [3]. Patients with CRSwNP complain of nasal obstruction, nasal discharge, and smell loss [4]. Bronchial inflammation and sinonasal inflammation are associated in comorbid subjects, and a significant correlation can be observed between the inflammatory patterns detectable in bronchial and nasal biopsies, thus further strengthening the pathogenic concept of united airway disease [5,6]. Moreover, a stronger association between asthma and CRSwNP occurs in patients with severe disease, as shown by a greater extent of airway inflammation, paralleled by worse bronchial obstruction and higher rates of nasal polyp recurrence [1]. Indeed, when CRSwNP is present in asthmatic patients, asthma is more difficult to control and is also characterized by an enhanced tendency to exacerbate [7]. Therefore, this observation suggests that CRSwNP can be a relevant risk factor for asthma severity [8]. On the other hand, patients with CRSwNP and concomitant asthma experience worse sinonasal symptoms such as nasal congestion and smell loss [1]. In comparison with asthmatic children, CRSwNP occurs much more frequently as a comorbidity of adult-onset asthma, thereby implying that the inflammatory changes associated with aging (inflammaging) play a key role in disease pathobiology [9].

**Figure 1:**
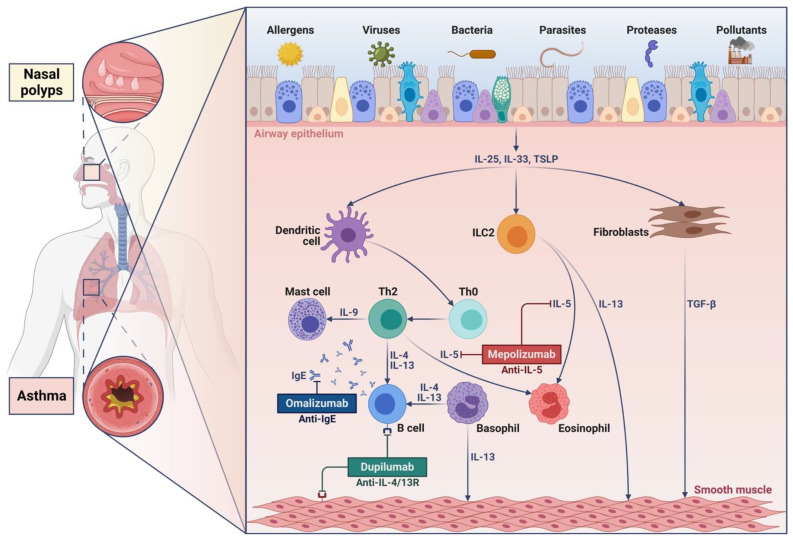
Pathobiologic mechanisms underlying type 2 airway inflammation in asthma and nasal polyposis. Released by damaged airway epithelium, alarmins (IL-25, IL-33, TSLP) activate dendritic cells, ILC2, and airway fibroblasts. As a consequence, an overproduction of type 2 cytokines (IL-4, IL-5, IL-13) occurs within both upper and lower airways, being responsible for the development of nasal polyposis and asthma. In addition to proinflammatory features, the type 2 trait also includes structural changes underpinning airway remodeling mediated by TGF-β and other growth factors. TSLP: thymic stromal lymphopoietin; Th: T helper; ILC2: group 2 innate lymphoid cells; IL: interleukin; TGF-β: transforming growth factor-β. This original figure was created by the authors using “BioRender.com”.

Asthma and CRSwNP are often driven by chronic type 2 inflammation, sustained by close interactions between innate and adaptive immune responses (Figure 1) [10]. The most important immune-inflammatory cells which orchestrate and coordinate upper and lower type 2 airway inflammation are T helper 2 (Th2) lymphocytes and group 2 innate lymphoid cells (ILC2) [2]. Other cells participating in the pathophysiologic process underlying type 2 asthma and CRSwNP include airway epithelial cells, dendritic cells, B lymphocytes, eosinophils, basophils, mast cells, and macrophages [2,10,11]. As a result, type 2 mechanisms can lead to the development, persistence, and amplification of a predominant eosinophilic endotype, associated or not with an immunoglobulin E (IgE)-mediated allergic phenotype [12,13,14]. Indeed, most patients with CRSwNP and asthma present in their nasal polyp tissue a marked eosinophilic infiltration possibly associated with elevated IgE levels, as well as with high eosinophil counts in both blood and sputum [4,15,16]. Patients with CRSwNP do not mount IgE-driven immune reactions only against inhaled allergens, but also versus *Staphylococcus aureus*-derived enterotoxins [1]. In particular, a large majority of these subjects have been shown to implement in their nasal polyp tissue a local production of polyclonal IgE directed towards staphylococcal enterotoxins [1,15]. Similarly, IgE targeting *S. aureus* enterotoxins can also contribute to the pathogenesis of severe asthma [17,18].

In addition to CRSwNP, asthma is also associated with nasal polyps in patients suffering from aspirin-exacerbated respiratory disease (AERD) [1], and it has been established that about 8–26% of subjects with CRSwNP complain of AERD [19,20]. AERD is characterized by the typical triad including asthma, nasal polyposis, and respiratory reactions to aspirin and other cyclooxygenase-1 inhibitors [21]. The development and recurrence of AERD are induced by type 2 respiratory inflammation, based on a complex interplay involving several cells and proinflammatory mediators. In particular, AERD is a non-allergic type 2 disease, characterized by mast cell degranulation, cysteinyl leukotriene overproduction, and platelet activation [21].

In asthma and nasal polyposis, type 2 pathomechanisms are responsible not only for upper and lower airway inflammation, but also for relevant structural changes. Within this context, bronchial remodeling is characterized by epithelial disruption, goblet cell hyperplasia, subepithelial fibrosis, smooth muscle thickening, and increased vascularization, whereas CRSwNP is marked by stromal edema, turbinate hypertrophy, collagen/fibrin deposition, and polyp formation [2,22,23,24,25,26].

Notably, the recent advances in the common pathophysiologic processes underpinning asthma and nasal polyposis highlight the therapeutic importance of targeting shared pathogenic cells and molecules, including eosinophils, IgE, type 2 cytokines, and their receptors (Figure 1) [27,28].

On the basis of the above concepts, the purpose of this narrative review is to discuss the pathobiologic mechanisms implicated in type 2 inflammation responsible for asthma and nasal polyposis, as well as to outline the therapeutic impact of the current biologic treatments which can be used to manage the most severe forms of both these diseases.

## 2. Cellular and Molecular Mechanisms Underlying Type 2 Inflammation in Asthma and Nasal Polyposis

Type 2 inflammation is the predominant pathologic trait underpinning asthma and nasal polyposis, driven by either allergic or non-allergic mechanisms [2]. In particular, in both upper and lower airways, type 2 inflammatory responses are triggered, maintained, and amplified by synergistic interactions between the innate and adaptive branches of the immune system, mainly mediated by group 2 innate lymphoid cells (ILC2) and T helper 2 (Th2) lymphocytes, which produce and secrete type 2 cytokines such as interleukins 4 (IL-4), 13 (IL-13), and 5 (IL-5) (Figure 1) [10,14,25,29,30,31,32]. Other cellular sources of type 2 cytokines include tissue-resident memory T cells (Trm), T follicular helper 2 (Tfh2) and 13 (Tfh13) cells, mast cells, basophils, and eosinophils [11,33,34,35,36]. Within this pathologic landscape, a key role is played by the dysregulation of airway epithelium, promoted by deleterious agents such as aeroallergens, airborne pollutants, smoking, and viral and bacterial infections, which damage both bronchial epithelial cells and sinonasal epithelial cells, thus stimulating their production of innate cytokines named alarmins [37,38,39]. These include thymic stromal lymphopoietin (TSLP), interleukin-25 (IL-25), and interleukin-33 (IL-33), which act as upstream triggers of innate and adaptive immune mechanisms underlying type 2 inflammation in both upper and lower airways (Figure 1) [37]. In particular, alarmins directly stimulate ILC2 and also induce dendritic cells to drive the differentiation of Th2 lymphocytes [25,31,40]. Alarmins recruit and activate ILC2 not only in patients with asthma and CRSwNP, but also in subjects with AERD [21]. Upon alarmin-dependent activation, ILC2 and Th2 cells, as well as mast cells, basophils, and eosinophils, secrete high amounts of proinflammatory mediators including IL-4, IL-13, IL-5, prostaglandin D_2_ (PGD_2_), and cysteinyl leukotrienes.

### 2.1. Interleukin-4 and Interleukin-13

IL-4 acts as an essential driver of the commitment of naïve CD4^+^ T lymphocytes towards the Th2 immunophenotype [41,42]. This crucial role of IL-4 is also facilitated by its capability of suppressing the immunomodulatory function of regulatory T (Treg) cells, which normally inhibit the differentiation of Th2 lymphocytes in healthy subjects [43,44]. In allergic patients, both IL-4 and IL-13 are responsible for immunoglobulin gene rearrangement at the level of B lymphocytes, thus inducing isotype switching and the consequent IgE synthesis [45,46]. The allergen-induced bridging of adjacent IgE bound to their high affinity receptors (FcεRI) expressed by mast cells and basophils (cross-linking) elicits the IgE-dependent degranulation of these cells, which further amplifies type 2 airway inflammation by increasing the production of IL-4 and IL-13 [40,47].

In addition to T and B lymphocytes, IL-4 and IL-13 also target other immune/inflammatory and resident cells of the airways. In regard to mast cells, IL-13 enhances cellular proliferation and FcεRI expression [48]. Furthermore, IL-4 and IL-13 activate the M2 macrophage subtype in patients with either asthma and/or CRSwNP [10,49,50,51]. IL-4 and IL-13 are also involved in eosinophil trafficking. Indeed, IL-4 increases the endothelial expression of the vascular cell adhesion molecule-1 (VCAM-1), thereby inducing eosinophil extravasation, whilst IL-13 promotes eosinophil chemotaxis by stimulating the release of eotaxin from airway epithelial cells [52,53]. Hence, IL-4 and IL-13 contribute remarkably to the massive eosinophil infiltration which often characterizes asthmatic bronchial walls, as well as the nasal polyps of patients with either CRSwNP or AERD [21,54].

With regard to airway resident cells, the epithelial barrier can be broken by IL-13 and IL-4 at both bronchial and sinonasal levels. In particular, IL-13 down-regulates the production of claudin-18.1, a key component of intercellular tight junctions [55]. Moreover, IL-4 and IL-13 up-regulate the biosynthesis of histone deacetylases 1 and 9 (HDAC 1 and 9), whose expression levels are directly correlated to the structural damage of airway epithelium [56]. By compromising the integrity of the sinonasal and bronchial epithelial layers in patients with asthma and nasal polyposis, IL-4 and IL-13 cause a dysfunction of the airway epithelial barriers, thus increasing their permeability to aeroallergens and infectious agents [57]. IL-13 induces goblet cell hyperplasia and stimulates the production of mucin 5AC (MUC5AC), a glycoprotein which enhances the viscosity of bronchial mucus and impairs the mucociliary clearance of nasal epithelium [58,59]. This biological action of IL-13 significantly contributes to mucus plugging, which is closely tied to type 2 inflammation and airway eosinophilic infiltration [60]. Within this pathogenic context, a key role is played by the very tight adhesion of mucus to the airway epithelium [61]. IL-13 also rises the airway epithelial expression of the inducible isoform of NO synthase (iNOS), thereby incrementing the levels of fractional exhaled nitric oxide (FeNO) [62], which is used in clinical practice as a reliable biomarker of airway inflammation. Other cellular targets of IL-13 are airway smooth muscle cells, whose contractile and proliferative responses can be induced by this type 2 cytokine [63]. Further contributions of IL-13 to airway remodeling include its biologic actions resulting in the stimulation of collagen deposition, fibroblast proliferation, sub-epithelial fibrotic thickening, and activation of the epithelial-mesenchymal trophic unit [10]. Such structural effects are at least in part mediated by the IL-13-dependent induction of transforming growth factor-β1 (TGF-β1), a fibrogenic mediator which crucially contributes to airway remodeling in asthma [64,65]. Airway remodeling elicited by IL-4 and IL-13 also occurs via their stimulatory action on M2-type macrophages, which promote fibrin deposition and nasal polyp formation by inhibiting fibrin degradation [64]. Indeed, these type 2 cytokines decrease fibrinolysis by reducing the biosynthesis of the tissue plasminogen activator [66]. In addition, the up-regulation of IL-13 expression in nasal polyp tissue is associated with the IL-13-induced increase in the number of M2 macrophages producing the coagulation factor XIIIA [51]. Therefore, these cytokines and their receptors represent suitable molecular targets for monoclonal antibodies (dupilumab) with the therapeutic potential of inhibiting airway remodeling in asthma and nasal polyposis [32].

### 2.2. Interleukin-5

The maturation, differentiation, proliferation, and activation of eosinophils are mostly attributable to IL-5, which also inhibits the apoptotic death of these cells [67,68,69]. In addition to ILC2 and Th2 cells, other cellular sources of IL-5 include mast cells, eosinophils themselves, and natural killer cells [68]. Furthermore, IL-5 induces the recruitment of eosinophils into the airways by acting synergistically with eotaxins, which are potent chemoattractants for these cells [70,71]. High serum concentrations of IL-5 can be detected in patients with severe asthma, even if eosinophilopoiesis takes place in these subjects not only in the bone marrow, but also within the airways [72]. Moreover, in asthmatic patients with the type 2 endotype, IL-5 stimulates the interaction between eosinophils and the matricellular protein periostin, whose levels increase when eosinophils infiltrate the airways [73]. IL-5 also promotes eosinophil degranulation [74], thereby contributing to the injury of both the bronchial epithelium and neural tissue via the release of cytotoxic proteins stored within cytoplasmic granules, including major basic protein, eosinophil cationic protein, eosinophil peroxidase, and eosinophil-derived neurotoxin [75,76]. In addition, when stimulated by IL-5, eosinophils are able to secrete TGF-β1 [77]. Upon IL-5 stimulation, eosinophils also activate a process named ETosis, which is based on the assembly and release of eosinophilic extracellular traps (EET), consisting of scaffold structures including granule proteins and mitochondrial DNA and responsible for the further worsening of airway inflammation in severe asthma [78,79].

High IL-5 levels and elevated counts of eosinophil progenitors and mature eosinophils can be detected in induced sputum obtained from allergic asthmatic patients [68]. Moreover, high concentrations of IL-5 and eotaxins were observed in induced sputum taken from subjects manifesting acute asthma exacerbations [80]. In induced sputum, IL-5 levels were found to be inversely correlated with apoptotic eosinophils in patients with either stable asthma or acute disease exacerbations [81,82]. Moreover, IL-5 expression is up-regulated in nasal polyp tissue [83], and high IL-5 levels correlate with more severe nasal polyposis [84]. Indeed, the local synthesis of IL-5 promotes the accumulation of eosinophils within the nasal mucosa of most patients with CRSwNP [85,86]. Furthermore, IL-5-dependent eosinophilic inflammation has been shown to significantly correlate with epithelial damage, smell loss, fibroblast activity, collagen production, and the deposition of fibrotic tissue within nasal polyps [87]. In this regard, it has been shown that molecular antibodies targeting IL-5 are able to disrupt IL-5-mediated intercellular networks involving eosinophils, mast cells, and airway epithelial cells, which drive nasal polyp development [88].

Therefore, IL-5 and its receptor appear to be proper therapeutic targets for biologic drugs (mepolizumab, reslizumab, benralizumab) that are able to inhibit eosinophilic inflammation and airway remodeling in both asthma and nasal polyposis [32].

## 3. Biological Therapies of Severe Asthma and Nasal Polyposis

Since the association between asthma and nasal polyposis in the same patient implies common underlying pathomechanisms shared by these two diseases, it is intuitive that currently available biologic therapies can be very useful to treat the inflammatory traits of both upper and lower airways. In particular, because type 2 inflammation is a frequent hallmark of severe asthma and CRSwNP, comorbid patients can significantly benefit from treatments targeting pathophysiologic pathways operated by several effector molecules including IgE, IL-5, and its receptor, as well as the receptors of IL-4 and IL-13 (Figure 1) [89]. Within this context, many randomized controlled trials (RCTs) and real-life studies have shown the therapeutic utility of omalizumab, mepolizumab, reslizumab, benralizumab, dupilumab, and tezepelumab [69]. Among these monoclonal antibodies, only omalizumab, mepolizumab, and dupilumab have been licensed for the biological treatment of both severe asthma and nasal polyposis.

### 3.1. Omalizumab

The humanized monoclonal antibody omalizumab binds to the two Cε3 domains of the constant region of human IgE, thus generating IgE/anti-IgE immune complexes which prevent IgE interactions with their high-affinity FcεRI and low-affinity FcεRII/CD23 receptors expressed by immune/inflammatory and airway structural cells [90,91,92]. Thanks to this mechanism of action, omalizumab has been shown to be very effective as an add-on biological therapy to severe allergic asthma, thus lowering the frequency of disease exacerbations and significantly improving lung function. Such findings derive from both RCTs and real-world investigations, and also document the long-lasting safety of omalizumab [93,94,95,96,97,98]. In particular, INNOVATE (INvestigatioN of Omalizumab in seVere Asthma TrEatment) was the key RCT which showed the above therapeutic effects, thus leading to the approval of omalizumab for the biological treatment of severe asthma [99]. Eligible patients for treatment with omalizumab must have serum IgE levels ranging from 30 to 1500 IU/mL, and they also need to be sensitized to perennial allergens.

Omalizumab is also capable of effectively and safely treating CRSwNP [100]. Indeed, the two POLYP-1 and POLYP-2 24-week phase 3 RCTs demonstrated that, in comparison with a placebo, omalizumab significantly improved nasal polyp score (NPS), nasal congestion score (NGS)m, and the 22-item sino-nasal outcome test (SNOT-22) score [101]. Moreover, a further 52-week extension trial carried out in patients who had already completed POLYP-1 or POLYP-2 showed the persistence of the efficacy and safety of omalizumab [102]. In this regard, the findings of a recent real-life study which reported that in patients with severe allergic asthma and CRSwNP omalizumab induced a significant improvement of both asthmatic and nasal symptoms are very interesting [103].

### 3.2. Mepolizumab

Mepolizumab is a humanized monoclonal IgG1/k antibody that specifically interacts with the α-chain of IL-5, thus inhibiting its binding to the α subunit of the IL-5 receptor (IL-5Rα) [104,105]. Several RCTs, including the DREAM (Dose Ranging Efficacy And safety with Mepolizumab), MENSA (MEpolizumab as adjunctive therapy iN patients with Severe Asthma), and SIRIUS (SteroId ReductIon with mepolizUmab Study) studies, proved that, in patients with severe eosinophilic asthma, mepolizumab decreased disease exacerbations and also improved quality of life, symptom control, and pulmonary function [106,107,108]. In addition, the SIRIUS trial showed that mepolizumab was able to significantly reduce the intake of oral corticosteroids (OCS) [108]. Indeed, this RCT was specifically designed to assess mepolizumab’s capability in decreasing OCS in patients with severe eosinophilic asthma undergoing chronic steroid treatment. The phase 3b MUSCA study further validated the effective therapeutic action of mepolizumab with regard to health-related quality of life [109]. Both COSMOS and COLUMBA studies positively evaluated the long-term efficacy and safety of mepolizumab [110,111]. Overall, these findings have been recently corroborated and extended by real-life observations, which also demonstrated that mepolizumab is effective in both allergic and non-allergic patients, improves lung function at the level of large and small airways, and can induce asthma remission [112,113,114,115,116,117,118]. The latter is a concept based on the ability of a given drug to decrease severe asthma exacerbations, improve symptom control, spare OCS use, and stabilize pulmonary function [119]. The required criteria for patient eligibility to mepolizumab include blood eosinophil levels of at least 150 cells/µL, associated with at least one blood count of 300 or more cells/µL during the previous 12 months. 

The phase 3, 52-week SYNAPSE trial showed that, in patients with CRSwNP, when compared with a placebo, mepolizumab induced significant improvements in both nasal obstruction assessed by a visual analogic scale (VAS) and total endoscopic nasal polyp score, as well as prolonged the time to the first needed nasal surgery [120]. These data convincingly confirmed similar results reported by a previous study performed in patients with recurrent nasal polyps: the experience of the good clinical effects of mepolizumab in regard to upper airway symptoms and the requirement of nasal surgery [121]. A recent Italian real-world clinical investigation has shown that, in patients with severe eosinophilic asthma and CRSwNP, mepolizumab decreased the blood eosinophil count and disease exacerbations, improved sino-nasal and bronchial symptoms, lowered OCS intake, and reduced the total endoscopic nasal polyp score [122].

### 3.3. Reslizumab

Reslizumab is another humanized IgG4/k monoclonal antibody which behaves as an IL-5 inhibitor [123,124]. Both phase 2 and phase 3 RCTs proved that, in patients with severe eosinophilic asthma, reslizumab decreased the frequency of disease exacerbations, as well as improved lung function at the level of the central and peripheral airways [125,126,127,128]. In particular, reslizumab seems to be especially effective in patients with severe eosinophilic, late onset asthma associated with CRSwNP [129]. The valuable therapeutic effects provided by reslizumab in severe eosinophilic asthmatics have also been confirmed in real-life clinical practice [130]. Differently from the other monoclonal antibodies, which are administered via the subcutaneous route, reslizumab must be given intravenously. Patient eligibility for reslizumab requires the presence of at least 400 eosinophils per µL of blood.

### 3.4. Benralizumab

The humanized and afucosylated IgG1/k monoclonal antibody benralizumab utilizes its Fab portions to occupy and blockade IL-5Rα, thus inhibiting the interaction between this receptor subunit and the natural ligand IL-5 [131]. Furthermore, the constant Fc fragment of benralizumab binds to the FcγRIIIa receptor expressed by natural killer (NK) cells, thereby inducing eosinophil death through antibody-dependent cell-mediated cytotoxicity (ADCC), a proapoptotic mechanism that is powerfully strengthened by antibody afucosylation [131,132].

Benralizumab has been extensively evaluated within the context of a wide program of RCTs named WINDWARD, including the phase 3 studies SIROCCO and CALIMA, which clearly showed that this biologic drug significantly lowered the number of exacerbations of severe eosinophilic asthma, and also had a positive impact on symptom control and lung function [133,134]. The improvement in pulmonary function was also confirmed by the phase 3 BISE trial, which demonstrated that benralizumab was capable of inducing a relevant increment of FEV_1_ (forced expiratory volume in one second) in subjects with eosinophilic asthma and blood eosinophil levels of at least 300 cells/μL [135]. Both ZONDA and PONENTE studies showed that benralizumab significantly decreased chronic OCS intake in patients with severe eosinophilic asthma [136,137]. Moreover, the phase 3 BORA extension trial documented the persistent good safety profile which characterizes the long-term use of benralizumab [138]. The results of RCTs have been further corroborated and extended by real-life investigations, which also reported that benralizumab is very effective in both allergic and non-allergic patients with severe eosinophilic asthma [139,140]. Such therapeutic properties make it possible to utilize benralizumab as a successful switch treatment for allergic subjects with severe eosinophilic asthma, who are partially unresponsive to omalizumab [141]. Taken together, the results of both RCTs and real-world observations characterize benralizumab as a monoclonal antibody capable of depleting blood eosinophils, decreasing asthma exacerbations and OCS intake, relieving symptoms, and also improving airflow limitation and alveolar air trapping [139,140,141,142,143,144,145]. Patient eligibility for treatment with benralizumab requires a blood eosinophil count of at least 300 cells/µL.

In addition to confirming many of the above findings, the phase 3b RCT ANDHI also showed that benralizumab improved the symptoms of nasal polyposis, as indicated by the significant reduction in SNOT-22 score [146]. In particular, an ANDHI sub-study aimed to evaluate the effects of benralizumab on upper airway symptoms, documented that the beneficial therapeutic action of this biologic drug was especially effective in those patients with severe eosinophilic asthma and nasal polyposis characterized by higher baseline SNOT-22 scores [146]. Moreover, some recent Italian real-life studies carried out in patients with severe eosinophilic asthma and nasal polyposis have shown that benralizumab can improve not only SNOT-22 score, but also endoscopic nasal polyp score and Lund–Mackay CT (computed tomography) scan score [147,148,149].

### 3.5. Dupilumab

Dupilumab binds to the α subunit of the IL-4 receptor (IL-4Rα) and prevents its dimerization with the other molecular components of both IL-4 (γC chain) and IL-13 (IL-13Rα1 subunit) receptors, thereby acting as a dual receptor antagonist of these two cytokines, whose biological effects are thus effectively inhibited by such a fully human IgG4 monoclonal antibody [150,151]. The two key phase 3 RCTs which led to the approval of dupilumab for add-on treatment of severe type 2 asthma were LIBERTY ASTHMA QUEST and LIBERTY ASTHMA VENTURE [152,153]. The first one recruited almost 2000 adult patients and showed that dupilumab, in comparison with a placebo, lowered the annualized rate of severe asthma exacerbations by about 50% [152]. This preventive effect rose to a more than 65% reduction when the blood eosinophil count amounted to at least 300 cells/μL. Furthermore, dupilumab ameliorated lung function by significantly increasing FEV_1_ and also improved the control of asthma symptoms, as well as decreasing the levels of relevant biomarkers of type 2 asthma such as FeNO and serum IgE concentrations [152]. The LIBERTY ASTHMA VENTURE study, specifically designed for patients undergoing chronic OCS treatment, demonstrated that dupilumab was very effective as OCS-sparing medication [153]. Indeed, according to this trial, dupilumab was able to lower and even zero OCS intake in a relevant percentage of the enrolled adult patients with severe type 2 asthma. In spite of the marked decrease in daily OCS consumption, dupilumab significantly decremented the number of severe asthma exacerbations by almost 60%, and also enhanced FEV_1_ by more than 200 mL [153]. Among the subjects who completed one of the above trials, many were enrolled in the TRAVERSE open-label extension study, which documented that the therapeutic effects of dupilumab observed in severe asthmatic patients were persistent and associated with a good safety and tolerability profile [154]. The clinical and functional positive findings detected in adult subjects were also confirmed by the phase 3 LIBERTY ASTHMA VOYAGE RCT in children (age: 6–11 years) with moderate-to-severe asthma [155]. In adults with severe asthma, the proven therapeutic effectiveness of dupilumab has also been verified by real-world investigations, showing that this monoclonal antibody can exert both short-term and long-lasting benefits [156,157,158,159]. Prescription criteria for dupilumab include blood eosinophil counts of at least 150 cells/µL and/or FeNO levels of at least 25 ppb.

With regard to the biologic therapy of nasal polyposis, some adult CRSwNP patients refractory to intranasal corticosteroids, with or without comorbid asthma, were enrolled in one of the first double-blind, randomized, and placebo-controlled studies, which showed that dupilumab bettered the SNOT-22 score, endoscopic nasal polyp score, Lund-Mackay CT score, and the sense of smell, evaluated by UPSIT (University of Pennsylvania Smell Identification Test) score [160]. Dupilumab has been licensed for treatment of CRSwNP thanks to two phase 3 RCTs named LIBERTY NP SINUS-24 and SINUS-52 [161]. These trials showed that dupilumab, when compared with a placebo, improved nasal obstruction and congestion, and also decreased the size of nasal polyps and the radiological opacification of paranasal sinuses, assessed through a Lund–Mackay CT score [161]. Moreover, the authors of a pooled analysis of these two studies reported that a relatively large number of asthmatic patients with nasal polyposis, treated with dupilumab, in comparison with a placebo experienced significant improvements in SNOT-22 and UPSIT scores [162]. These patients also manifested decreased needs for OCS use and sinonasal surgery [162].

## 4. Closing Remarks

Patients with asthma and nasal polyposis are frequently characterized by a predominant type 2 trait of airway inflammation (Figure 1). Type 2 inflammatory features mainly include dysfunction of the epithelial barrier and eosinophilic infiltration, often detectable at both bronchial and sinonasal levels and especially in cases of severe disease. Within such an airway context, type 2 asthma and nasal polyposis develop as a consequence of the overexpression of IL-4, IL-13, and IL-5. The exaggerated production of these cytokines is driven by two main cellular pathways, orchestrated and amplified by the innate and adaptive immunopathologic mechanisms sustained by ILC2 and Th2 lymphocytes, respectively. These proinflammatory networks can lead to the development of either allergic or non-allergic type 2 endotypes. Because of the common underlying pathomechanisms, severe asthma and nasal polyposis can be successfully treated together by the same monoclonal antibodies, targeting IgE, type 2 cytokines, or their receptors (Figure 1). Indeed, these biologic drugs can improve symptom control and airflow limitation at the level of both the lower and upper airways. 

## Data Availability

Not applicable.
